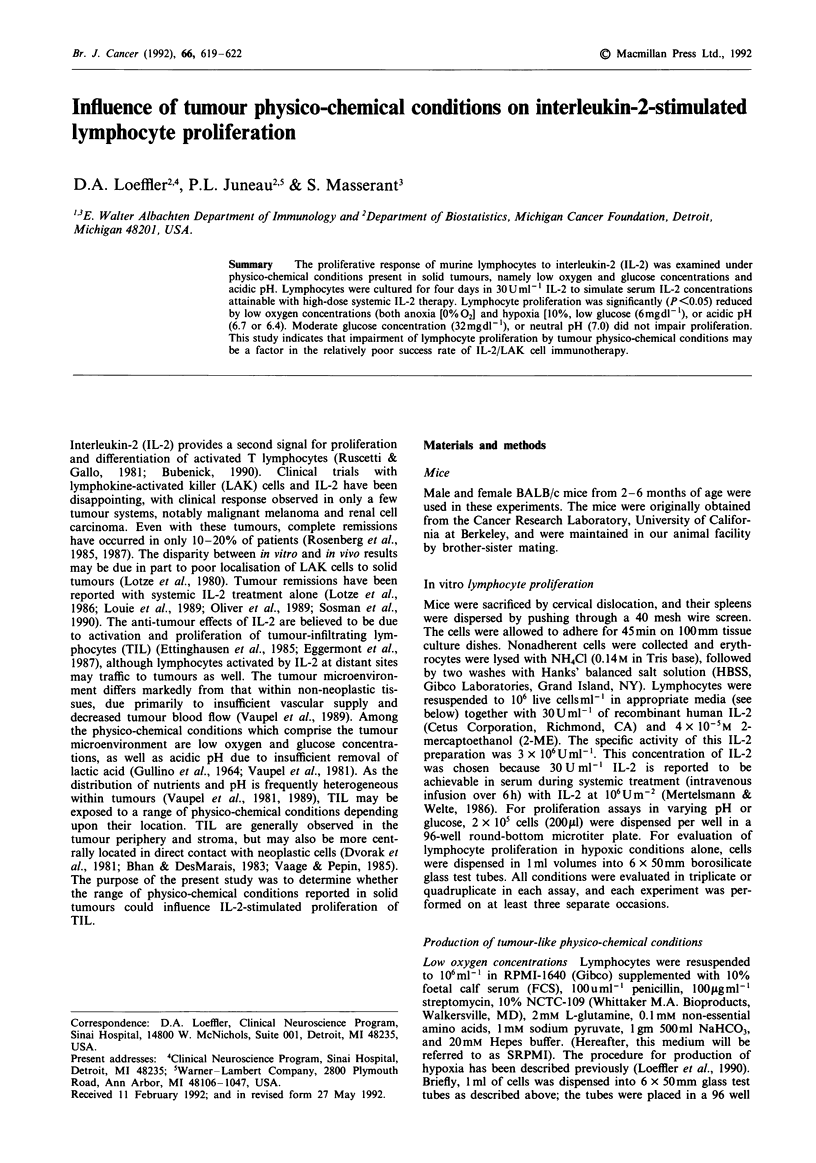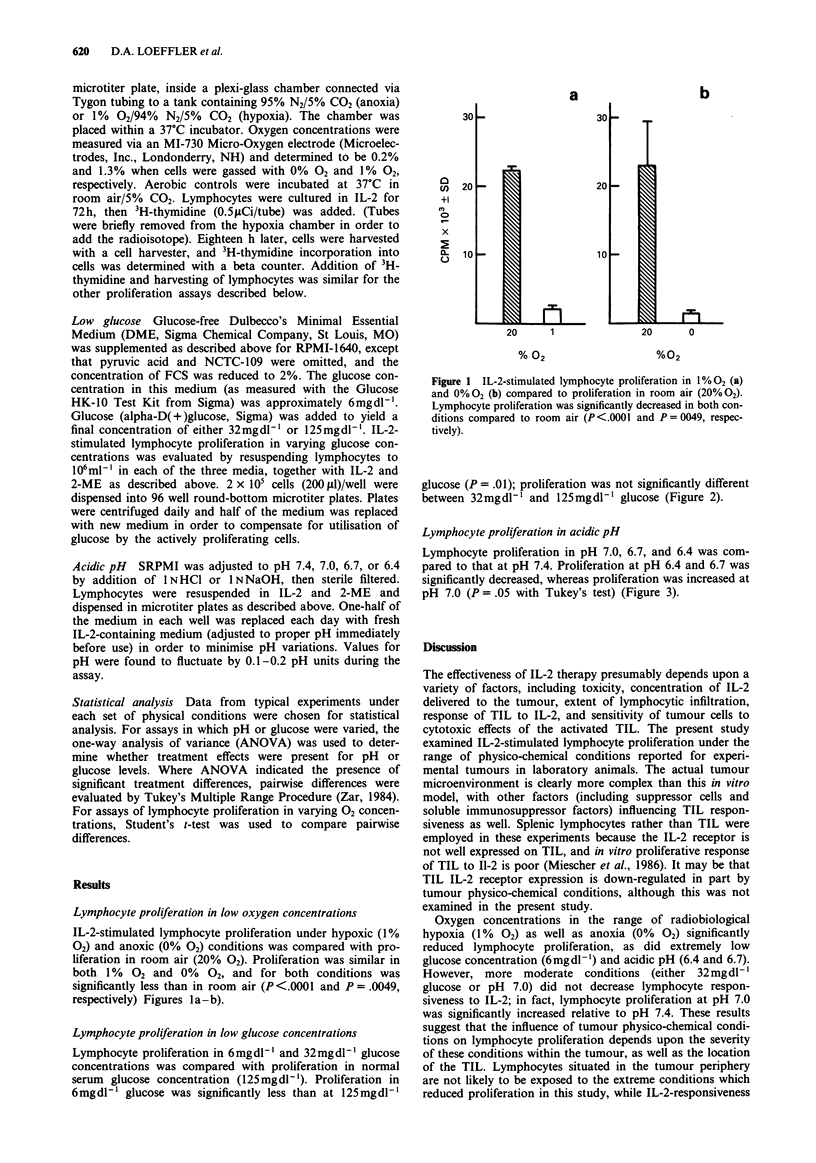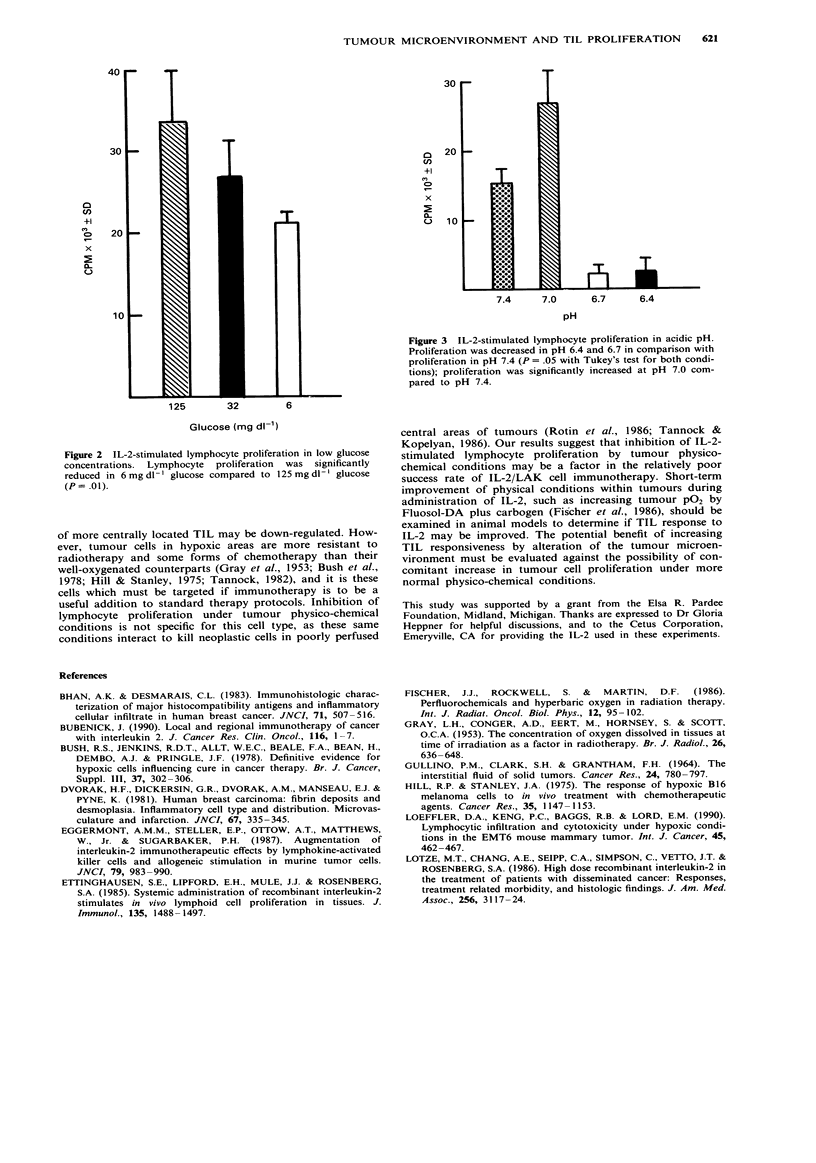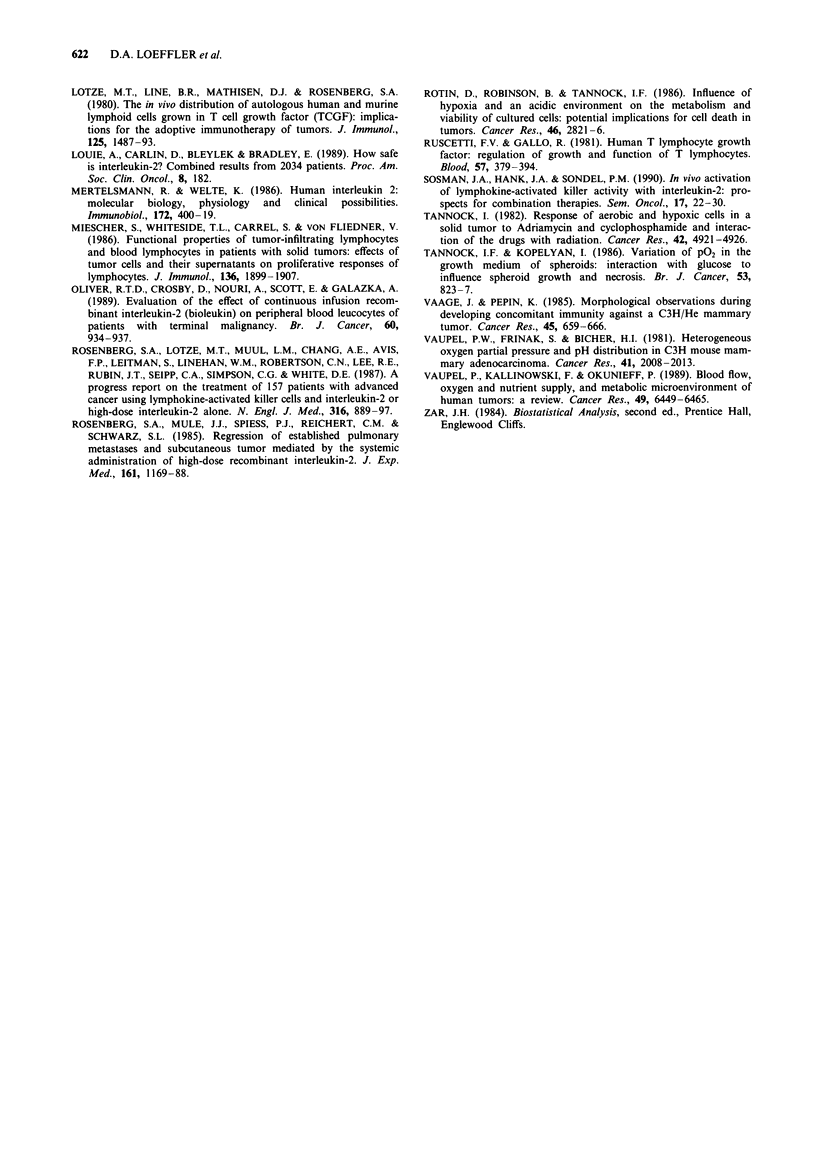# Influence of tumour physico-chemical conditions on interleukin-2-stimulated lymphocyte proliferation.

**DOI:** 10.1038/bjc.1992.326

**Published:** 1992-10

**Authors:** D. A. Loeffler, P. L. Juneau, S. Masserant

**Affiliations:** E. Walter Albachten Department of Immunology, Michigan Cancer Foundation, Detroit 48201.

## Abstract

The proliferative response of murine lymphocytes to interleukin-2 (IL-2) was examined under physico-chemical conditions present in solid tumours, namely low oxygen and glucose concentrations and acidic pH. Lymphocytes were cultured for four days in 30 U ml-1 IL-2 to simulate serum IL-2 concentrations attainable with high-dose systemic IL-2 therapy. Lymphocyte proliferation was significantly (P < 0.05) reduced by low oxygen concentrations (both anoxia [0% O2] and hypoxia [10%, low glucose (6 mg dl-1), or acidic pH (6.7 or 6.4). Moderate glucose concentration (32 mg dl-1), or neutral pH (7.0) did not impair proliferation. This study indicates that impairment of lymphocyte proliferation by tumour physico-chemical conditions may be a factor in the relatively poor success rate of IL-2/LAK cell immunotherapy.


Br. J. Cancer (1992), 66, 619–622 © Macmillan Press Ltd., 1992

### Influence of tumour physico-chemical conditions on interleukin-2-stimulated lymphocyte proliferation

D.A. Loeffler[2,4], P.L. Juneau[2,5] & S. Masserant[3]

[1,3]E. Walter Albachten Department of Immunology and [2]Department of Biostatistics, Michigan Cancer Foundation, Detroit, Michigan 48201, USA.

**Summary** The proliferative response of murine lymphocytes to interleukin-2 (IL-2) was examined under physico-chemical conditions present in solid tumours, namely low oxygen and glucose concentrations and acidic pH. Lymphocytes were cultured for four days in $30 \mathrm{U\,ml^{-1}}$ IL-2 to simulate serum IL-2 concentrations attainable with high-dose systemic IL-2 therapy. Lymphocyte proliferation was significantly ($P<0.05$) reduced by low oxygen concentrations (both anoxia [0% $O_2$] and hypoxia [10%, low glucose ($6 \mathrm{mg\,dl^{-1}}$), or acidic pH (6.7 or 6.4). Moderate glucose concentration ($32 \mathrm{mg\,dl^{-1}}$), or neutral pH (7.0) did not impair proliferation. This study indicates that impairment of lymphocyte proliferation by tumour physico-chemical conditions may be a factor in the relatively poor success rate of IL-2/LAK cell immunotherapy.

Interleukin-2 (IL-2) provides a second signal for proliferation and differentiation of activated T lymphocytes (Ruscetti & Gallo, 1981; Bubenick, 1990). Clinical trials with lymphokine-activated killer (LAK) cells and IL-2 have been disappointing, with clinical response observed in only a few tumour systems, notably malignant melanoma and renal cell carcinoma. Even with these tumours, complete remissions have occurred in only 10–20% of patients (Rosenberg *et al.*, 1985, 1987). The disparity between *in vitro* and *in vivo* results may be due in part to poor localisation of LAK cells to solid tumours (Lotze *et al.*, 1980). Tumour remissions have been reported with systemic IL-2 treatment alone (Lotze *et al.*, 1986; Louie *et al.*, 1989; Oliver *et al.*, 1989; Sosman *et al.*, 1990). The anti-tumour effects of IL-2 are believed to be due to activation and proliferation of tumour-infiltrating lymphocytes (TIL) (Ettinghausen *et al.*, 1985; Eggermont *et al.*, 1987), although lymphocytes activated by IL-2 at distant sites may traffic to tumours as well. The tumour microenvironment differs markedly from that within non-neoplastic tissues, due primarily to insufficient vascular supply and decreased tumour blood flow (Vaupel *et al.*, 1989). Among the physico-chemical conditions which comprise the tumour microenvironment are low oxygen and glucose concentrations, as well as acidic pH due to insufficient removal of lactic acid (Gullino *et al.*, 1964; Vaupel *et al.*, 1981). As the distribution of nutrients and pH is frequently heterogeneous within tumours (Vaupel *et al.*, 1981, 1989), TIL may be exposed to a range of physico-chemical conditions depending upon their location. TIL are generally observed in the tumour periphery and stroma, but may also be more centrally located in direct contact with neoplastic cells (Dvorak *et al.*, 1981; Bhan & DesMarais, 1983; Vaage & Pepin, 1985). The purpose of the present study was to determine whether the range of physico-chemical conditions reported in solid tumours could influence IL-2-stimulated proliferation of TIL.

Correspondence: D.A. Loeffler, Clinical Neuroscience Program, Sinai Hospital, 14800 W. McNichols, Suite 001, Detroit, MI 48235, USA.
Present addresses: [4]Clinical Neuroscience Program, Sinai Hospital, Detroit, MI 48235; [5]Warner-Lambert Company, 2800 Plymouth Road, Ann Arbor, MI 48106-1047, USA.
Received 11 February 1992; and in revised form 27 May 1992.

### Materials and methods

### Mice

Male and female BALB/c mice from 2–6 months of age were used in these experiments. The mice were originally obtained from the Cancer Research Laboratory, University of California at Berkeley, and were maintained in our animal facility by brother-sister mating.

### In vitro *lymphocyte proliferation*

Mice were sacrificed by cervical dislocation, and their spleens were dispersed by pushing through a 40 mesh wire screen. The cells were allowed to adhere for 45 min on 100 mm tissue culture dishes. Nonadherent cells were collected and erythrocytes were lysed with $NH_4Cl$ (0.14 M in Tris base), followed by two washes with Hanks' balanced salt solution (HBSS, Gibco Laboratories, Grand Island, NY). Lymphocytes were resuspended to $10^6$ live cells $\mathrm{ml^{-1}}$ in appropriate media (see below) together with $30 \mathrm{U\,ml^{-1}}$ of recombinant human IL-2 (Cetus Corporation, Richmond, CA) and $4 \times 10^{-5}$ M 2-mercaptoethanol (2-ME). The specific activity of this IL-2 preparation was $3 \times 10^6 \mathrm{U\,ml^{-1}}$. This concentration of IL-2 was chosen because $30 \mathrm{U\,ml^{-1}}$ IL-2 is reported to be achievable in serum during systemic treatment (intravenous infusion over 6 h) with IL-2 at $10^6 \mathrm{U\,m^{-2}}$ (Mertelsmann & Welte, 1986). For proliferation assays in varying pH or glucose, $2 \times 10^5$ cells (200 µl) were dispensed per well in a 96-well round-bottom microtiter plate. For evaluation of lymphocyte proliferation in hypoxic conditions alone, cells were dispensed in 1 ml volumes into 6 × 50 mm borosilicate glass test tubes. All conditions were evaluated in triplicate or quadruplicate in each assay, and each experiment was performed on at least three separate occasions.

### *Production of tumour-like physico-chemical conditions*

*Low oxygen concentrations* Lymphocytes were resuspended to $10^6 \mathrm{ml^{-1}}$ in RPMI-1640 (Gibco) supplemented with 10% foetal calf serum (FCS), $100 \mathrm{u\,ml^{-1}}$ penicillin, $100 \mathrm{\mu g\,ml^{-1}}$ streptomycin, 10% NCTC-109 (Whittaker M.A. Bioproducts, Walkersville, MD), 2 mM L-glutamine, 0.1 mM non-essential amino acids, 1 mM sodium pyruvate, 1 gm 500 ml $NaHCO_3$, and 20 mM Hepes buffer. (Hereafter, this medium will be referred to as SRPMI). The procedure for production of hypoxia has been described previously (Loeffler *et al.*, 1990). Briefly, 1 ml of cells was dispensed into 6 × 50 mm glass test tubes as described above; the tubes were placed in a 96 well microtiter plate, inside a plexi-glass chamber connected via Tygon tubing to a tank containing 95% $N_2$/5% $CO_2$ (anoxia) or 1% $O_2$/94% $N_2$/5% $CO_2$ (hypoxia). The chamber was placed within a 37°C incubator. Oxygen concentrations were measured via an MI-730 Micro-Oxygen electrode (Microelectrodes, Inc., Londonderry, NH) and determined to be 0.2% and 1.3% when cells were gassed with 0% $O_2$ and 1% $O_2$, respectively. Aerobic controls were incubated at 37°C in room air/5% $CO_2$. Lymphocytes were cultured in IL-2 for 72h, then $^3$H-thymidine (0.5μCi/tube) was added. (Tubes were briefly removed from the hypoxia chamber in order to add the radioisotope). Eighteen h later, cells were harvested with a cell harvester, and $^3$H-thymidine incorporation into cells was determined with a beta counter. Addition of $^3$H-thymidine and harvesting of lymphocytes was similar for the other proliferation assays described below.

*Low glucose* Glucose-free Dulbecco's Minimal Essential Medium (DME, Sigma Chemical Company, St Louis, MO) was supplemented as described above for RPMI-1640, except that pyruvic acid and NCTC-109 were omitted, and the concentration of FCS was reduced to 2%. The glucose concentration in this medium (as measured with the Glucose HK-10 Test Kit from Sigma) was approximately 6mgdl$^{-1}$. Glucose (alpha-D(+)glucose, Sigma) was added to yield a final concentration of either 32mgdl$^{-1}$ or 125mgdl$^{-1}$. IL-2-stimulated lymphocyte proliferation in varying glucose concentrations was evaluated by resuspending lymphocytes to $10^6$ml$^{-1}$ in each of the three media, together with IL-2 and 2-ME as described above. $2 \times 10^5$ cells (200 μl)/well were dispensed into 96 well round-bottom microtiter plates. Plates were centrifuged daily and half of the medium was replaced with new medium in order to compensate for utilisation of glucose by the actively proliferating cells.

*Acidic pH* SRPMI was adjusted to pH 7.4, 7.0, 6.7, or 6.4 by addition of 1 NHCl or 1 NNaOH, then sterile filtered. Lymphocytes were resuspended in IL-2 and 2-ME and dispensed in microtiter plates as described above. One-half of the medium in each well was replaced each day with fresh IL-2-containing medium (adjusted to proper pH immediately before use) in order to minimise pH variations. Values for pH were found to fluctuate by 0.1–0.2 pH units during the assay.

*Statistical analysis* Data from typical experiments under each set of physical conditions were chosen for statistical analysis. For assays in which pH or glucose were varied, the one-way analysis of variance (ANOVA) was used to determine whether treatment effects were present for pH or glucose levels. Where ANOVA indicated the presence of significant treatment differences, pairwise differences were evaluated by Tukey's Multiple Range Procedure (Zar, 1984). For assays of lymphocyte proliferation in varying $O_2$ concentrations, Student's *t*-test was used to compare pairwise differences.

### Results

*Lymphocyte proliferation in low oxygen concentrations*

IL-2-stimulated lymphocyte proliferation under hypoxic (1% $O_2$) and anoxic (0% $O_2$) conditions was compared with proliferation in room air (20% $O_2$). Proliferation was similar in both 1% $O_2$ and 0% $O_2$, and for both conditions was significantly less than in room air ($P < .0001$ and $P = .0049$, respectively) Figures 1a–b).

*Lymphocyte proliferation in low glucose concentrations*

Lymphocyte proliferation in 6mgdl$^{-1}$ and 32mgdl$^{-1}$ glucose concentrations was compared with proliferation in normal serum glucose concentration (125mgdl$^{-1}$). Proliferation in 6mgdl$^{-1}$ glucose was significantly less than at 125mgdl$^{-1}$ glucose ($P = .01$); proliferation was not significantly different between 32mgdl$^{-1}$ and 125mgdl$^{-1}$ glucose (Figure 2).

**Figure 1** IL-2-stimulated lymphocyte proliferation in 1% $O_2$ (**a**) and 0% $O_2$ (**b**) compared to proliferation in room air (20% $O_2$). Lymphocyte proliferation was significantly decreased in both conditions compared to room air ($P < .0001$ and $P = 0049$, respectively).

*Lymphocyte proliferation in acidic pH*

Lymphocyte proliferation in pH 7.0, 6.7, and 6.4 was compared to that at pH 7.4. Proliferation at pH 6.4 and 6.7 was significantly decreased, whereas proliferation was increased at pH 7.0 ($P = .05$ with Tukey's test) (Figure 3).

### Discussion

The effectiveness of IL-2 therapy presumably depends upon a variety of factors, including toxicity, concentration of IL-2 delivered to the tumour, extent of lymphocytic infiltration, response of TIL to IL-2, and sensitivity of tumour cells to cytotoxic effects of the activated TIL. The present study examined IL-2-stimulated lymphocyte proliferation under the range of physico-chemical conditions reported for experimental tumours in laboratory animals. The actual tumour microenvironment is clearly more complex than this *in vitro* model, with other factors (including suppressor cells and soluble immunosuppressor factors) influencing TIL responsiveness as well. Splenic lymphocytes rather than TIL were employed in these experiments because the IL-2 receptor is not well expressed on TIL, and *in vitro* proliferative response of TIL to Il-2 is poor (Miescher *et al.*, 1986). It may be that TIL IL-2 receptor expression is down-regulated in part by tumour physico-chemical conditions, although this was not examined in the present study.

Oxygen concentrations in the range of radiobiological hypoxia (1% $O_2$) as well as anoxia (0% $O_2$) significantly reduced lymphocyte proliferation, as did extremely low glucose concentration (6mgdl$^{-1}$) and acidic pH (6.4 and 6.7). However, more moderate conditions (either 32mgdl$^{-1}$ glucose or pH 7.0) did not decrease lymphocyte responsiveness to IL-2; in fact, lymphocyte proliferation at pH 7.0 was significantly increased relative to pH 7.4. These results suggest that the influence of tumour physico-chemical conditions on lymphocyte proliferation depends upon the severity of these conditions within the tumour, as well as the location of the TIL. Lymphocytes situated in the tumour periphery are not likely to be exposed to the extreme conditions which reduced proliferation in this study, while IL-2-responsiveness

**Figure 2** IL-2-stimulated lymphocyte proliferation in low glucose concentrations. Lymphocyte proliferation was significantly reduced in 6 mg $dl^{-1}$ glucose compared to 125 mg $dl^{-1}$ glucose ($P = .01$).

**Figure 3** IL-2-stimulated lymphocyte proliferation in acidic pH. Proliferation was decreased in pH 6.4 and 6.7 in comparison with proliferation in pH 7.4 ($P = .05$ with Tukey's test for both conditions); proliferation was significantly increased at pH 7.0 compared to pH 7.4.

of more centrally located TIL may be down-regulated. However, tumour cells in hypoxic areas are more resistant to radiotherapy and some forms of chemotherapy than their well-oxygenated counterparts (Gray *et al.*, 1953; Bush *et al.*, 1978; Hill & Stanley, 1975; Tannock, 1982), and it is these cells which must be targeted if immunotherapy is to be a useful addition to standard therapy protocols. Inhibition of lymphocyte proliferation under tumour physico-chemical conditions is not specific for this cell type, as these same conditions interact to kill neoplastic cells in poorly perfused central areas of tumours (Rotin *et al.*, 1986; Tannock & Kopelyan, 1986). Our results suggest that inhibition of IL-2-stimulated lymphocyte proliferation by tumour physico-chemical conditions may be a factor in the relatively poor success rate of IL-2/LAK cell immunotherapy. Short-term improvement of physical conditions within tumours during administration of IL-2, such as increasing tumour $pO_2$ by Fluosol-DA plus carbogen (Fischer *et al.*, 1986), should be examined in animal models to determine if TIL response to IL-2 may be improved. The potential benefit of increasing TIL responsiveness by alteration of the tumour microenvironment must be evaluated against the possibility of concomitant increase in tumour cell proliferation under more normal physico-chemical conditions.

This study was supported by a grant from the Elsa R. Pardee Foundation, Midland, Michigan. Thanks are expressed to Dr Gloria Heppner for helpful discussions, and to the Cetus Corporation, Emeryville, CA for providing the IL-2 used in these experiments.

### References

BHAN, A.K. & DESMARAIS, C.L. (1983). Immunohistologic characterization of major histocompatibility antigens and inflammatory cellular infiltrate in human breast cancer. *JNCI*, **71**, 507–516.

BUBENICK, J. (1990). Local and regional immunotherapy of cancer with interleukin 2. *J. Cancer Res. Clin. Oncol.*, **116**, 1–7.

BUSH, R.S., JENKINS, R.D.T., ALLT, W.E.C., BEALE, F.A., BEAN, H., DEMBO, A.J. & PRINGLE, J.F. (1978). Definitive evidence for hypoxic cells influencing cure in cancer therapy. *Br. J. Cancer*, Suppl. III, **37**, 302–306.

DVORAK, H.F., DICKERSIN, G.R., DVORAK, A.M., MANSEAU, E.J. & PYNE, K. (1981). Human breast carcinoma: fibrin deposits and desmoplasia. Inflammatory cell type and distribution. Microvasculature and infarction. *JNCI*, **67**, 335–345.

EGGERMONT, A.M.M., STELLER, E.P., OTTOW, A.T., MATTHEWS, W., Jr. & SUGARBAKER, P.H. (1987). Augmentation of interleukin-2 immunotherapeutic effects by lymphokine-activated killer cells and allogeneic stimulation in murine tumor cells. *JNCI*, **79**, 983–990.

ETTINGHAUSEN, S.E., LIPFORD, E.H., MULE, J.J. & ROSENBERG, S.A. (1985). Systemic administration of recombinant interleukin-2 stimulates *in vivo* lymphoid cell proliferation in tissues. *J. Immunol.*, **135**, 1488–1497.

FISCHER, J.J., ROCKWELL, S. & MARTIN, D.F. (1986). Perfluorochemicals and hyperbaric oxygen in radiation therapy. *Int. J. Radiat. Oncol. Biol. Phys.*, **12**, 95–102.

GRAY, L.H., CONGER, A.D., EERT, M., HORNSEY, S. & SCOTT, O.C.A. (1953). The concentration of oxygen dissolved in tissues at time of irradiation as a factor in radiotherapy. *Br. J. Radiol.*, **26**, 636–648.

GULLINO, P.M., CLARK, S.H. & GRANTHAM, F.H. (1964). The interstitial fluid of solid tumors. *Cancer Res.*, **24**, 780–797.

HILL, R.P. & STANLEY, J.A. (1975). The response of hypoxic B16 melanoma cells to *in vivo* treatment with chemotherapeutic agents. *Cancer Res.*, **35**, 1147–1153.

LOEFFLER, D.A., KENG, P.C., BAGGS, R.B. & LORD, E.M. (1990). Lymphocytic infiltration and cytotoxicity under hypoxic conditions in the EMT6 mouse mammary tumor. *Int. J. Cancer*, **45**, 462–467.

LOTZE, M.T., CHANG, A.E., SEIPP, C.A., SIMPSON, C., VETTO, J.T. & ROSENBERG, S.A. (1986). High dose recombinant interleukin-2 in the treatment of patients with disseminated cancer: Responses, treatment related morbidity, and histologic findings. *J. Am. Med. Assoc.*, **256**, 3117–24.

LOTZE, M.T., LINE, B.R., MATHISEN, D.J. & ROSENBERG, S.A. (1980). The *in vivo* distribution of autologous human and murine lymphoid cells grown in T cell growth factor (TCGF): implications for the adoptive immunotherapy of tumors. *J. Immunol.*, **125**, 1487-93.

LOUIE, A., CARLIN, D., BLEYLEK & BRADLEY, E. (1989). How safe is interleukin-2? Combined results from 2034 patients. *Proc. Am. Soc. Clin. Oncol.*, **8**, 182.

MERTELSMANN, R. & WELTE, K. (1986). Human interleukin 2: molecular biology, physiology and clinical possibilities. *Immunobiol.*, **172**, 400-19.

MIESCHER, S., WHITESIDE, T.L., CARREL, S. & VON FLIEDNER, V. (1986). Functional properties of tumor-infiltrating lymphocytes and blood lymphocytes in patients with solid tumors: effects of tumor cells and their supernatants on proliferative responses of lymphocytes. *J. Immunol.*, **136**, 1899-1907.

OLIVER, R.T.D., CROSBY, D., NOURI, A., SCOTT, E. & GALAZKA, A. (1989). Evaluation of the effect of continuous infusion recombinant interleukin-2 (bioleukin) on peripheral blood leucocytes of patients with terminal malignancy. *Br. J. Cancer*, **60**, 934-937.

ROSENBERG, S.A., LOTZE, M.T., MUUL, L.M., CHANG, A.E., AVIS, F.P., LEITMAN, S., LINEHAN, W.M., ROBERTSON, C.N., LEE, R.E., RUBIN, J.T., SEIPP, C.A., SIMPSON, C.G. & WHITE, D.E. (1987). A progress report on the treatment of 157 patients with advanced cancer using lymphokine-activated killer cells and interleukin-2 or high-dose interleukin-2 alone. *N. Engl. J. Med.*, **316**, 889-97.

ROSENBERG, S.A., MULE, J.J., SPIESS, P.J., REICHERT, C.M. & SCHWARZ, S.L. (1985). Regression of established pulmonary metastases and subcutaneous tumor mediated by the systemic administration of high-dose recombinant interleukin-2. *J. Exp. Med.*, **161**, 1169-88.

ROTIN, D., ROBINSON, B. & TANNOCK, I.F. (1986). Influence of hypoxia and an acidic environment on the metabolism and viability of cultured cells: potential implications for cell death in tumors. *Cancer Res.*, **46**, 2821-6.

RUSCETTI, F.V. & GALLO, R. (1981). Human T lymphocyte growth factor: regulation of growth and function of T lymphocytes. *Blood*, **57**, 379-394.

SOSMAN, J.A., HANK, J.A. & SONDEL, P.M. (1990). *In vivo* activation of lymphokine-activated killer activity with interleukin-2: prospects for combination therapies. *Sem. Oncol.*, **17**, 22-30.

TANNOCK, I. (1982). Response of aerobic and hypoxic cells in a solid tumor to Adriamycin and cyclophosphamide and interaction of the drugs with radiation. *Cancer Res.*, **42**, 4921-4926.

TANNOCK, I.F. & KOPELYAN, I. (1986). Variation of $pO_2$ in the growth medium of spheroids: interaction with glucose to influence spheroid growth and necrosis. *Br. J. Cancer*, **53**, 823-7.

VAAGE, J. & PEPIN, K. (1985). Morphological observations during developing concomitant immunity against a C3H/He mammary tumor. *Cancer Res.*, **45**, 659-666.

VAUPEL, P.W., FRINAK, S. & BICHER, H.I. (1981). Heterogeneous oxygen partial pressure and pH distribution in C3H mouse mammary adenocarcinoma. *Cancer Res.*, **41**, 2008-2013.

VAUPEL, P., KALLINOWSKI, F. & OKUNIEFF, P. (1989). Blood flow, oxygen and nutrient supply, and metabolic microenvironment of human tumors: a review. *Cancer Res.*, **49**, 6449-6465.

ZAR, J.H. (1984). *Biostatistical Analysis*, second ed., Prentice Hall, Englewood Cliffs.